# Creating a novel strategy to reduce unnecessary laboratory testing based on healthcare cost analysis in high‐risk pregnancies and delivery ward

**DOI:** 10.1002/jcla.23759

**Published:** 2021-03-21

**Authors:** Mahboobeh Shirazi, Yusuf Masoudian, Elham Feizabad, Fatemeh Golshahi, Marjan Ghaemi

**Affiliations:** ^1^ Department of Obstetrics and Gynecology Yas Hospital Tehran University of Medical Sciences Tehran Iran; ^2^ Maternal, Fetal & Neonatal Research Centre Tehran University of Medical Sciences Tehran Iran; ^3^ Vali‐e‐Asr Reproductive Health Research Center, (VRHRC) Tehran University of Medical Sciences Tehran Iran

**Keywords:** cost analysis, clinical laboratory services, education, residency, obstetrics, education

## Abstract

**Introduction:**

Inappropriate request for laboratory tests is a challenging problem and an important cause for additional healthcare costs. Indeed, it may have further ambiguity for the clinicians. This study aimed to design an education‐based program to reduce unnecessary laboratory testing orders and the associated costs.

**Materials and methods:**

In this interventional prospective study that took place in an educational hospital, the type and frequency of selected laboratory testing requested by gynecology, and obstetrics residents in the patients with gestational diabetes mellitus, preeclampsia, preterm labor, and premature preterm rupture of the membrane as well as cesarean section and normal vaginal delivery were analyzed periodically in a 1‐year interval. At the same time, continuous educational supports and monitoring were performed. The results were compared before and after interventions.

**Results:**

The educational intervention regardless of the etiologies of the admission, decreased the requested laboratory testing significantly (*p* < 0.001), except for CBC. Indeed, no near misses or delays in treatment were observed. Cost analysis showed a 31.3% reduction of expenses per inpatient day due to the decrease in the number of daily laboratory testing ordered.

**Conclusions:**

Appropriate education and continuous monitoring of the residents could reduce the unrequired laboratory testing as well as healthcare costs.

## Graphical abstract

- Clinical decisions based on the principles of health economics play an important role in reducing costs.
- Appropriate education and continuous monitoring of the residents could reduce the unnecessary laboratory tests.
- In this regard, the design and initiation of electronic laboratory facilities to record and prevent the unnecessary laboratory test is mandatory.
- In‐hospital collaboration is essential to designing a suitable guideline for residents.

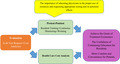

## 1 INTRODUCTION

Inappropriate clinical laboratory testing is a common problem in hospitals that may be due to practice variation and practice guidelines.^1^ Thus, unnecessary laboratory testing led to huge money loss and false‐positive results^2^ (as known as a diagnostic cascade),^3^ overdiagnosis, and overtreatment.^4^ Indeed, it may cause iatrogenic anemia and negative impacts on patient experience.^5^ Furthermore, discussing the unnecessary laboratory results for patients is very time‐consuming.^6^

Successful educational training of the residents is a primary goal in the teaching hospitals.^7^ In reviewing the performance of the residents, the number of tests and repetition were both high.^8^ On the other hand, the attempts to encourage clinicians to request the laboratory test more consciously are not successful.^9^

The main approach to decrease the rate of non‐required laboratory tests is an appropriate education and periodical reminder for the clinicians.^10^, ^11^ The effects of such education programs can be varied according to the ward and also by various training methods.^12^, ^13^, ^14^

The overall attempts to stop unnecessary laboratory testing have not been documented well, although some efforts have performed to reduce this amount.^8^ It should be noted that advising the clinicians for proper laboratory test requests may be effective but not sufficient.^15^ It seems that staff training often lacks sustainability after stopping the program that shows the need for continuous training.^16^ The ultimate goal of this study is to evaluate the effect of a continuous educational program in the residents to be adherence to the standard protocols in laboratory test ordering.

## 2 MATERIALS AND METHODS

### 2.1 Study setting and design

This interventional study was taken place in Yas hospital a tertiary educational center affiliated to Tehran University of Medical Sciences (TUMS), Tehran, Iran from March 2019 to March 2020.

For the initial coordination, a meeting was organized with the deputy director of education, the person in charge of quality improvement, the heads of clinical departments and the director of the laboratory and the study goals were described.

The laboratory data (type and frequency) requested by residents (under the guide of their supervisors) in the obstetrics and labor ward were analyzed. The demographic and clinical data of the patients such as age and admitting indication were collected either. The records of the patients admitted in intensive care unit or emergent situation were not collected, because these situations may alter the order set.

The knowledge of the residents about the type and frequency of required laboratory testing as well as the cost of each test assessed. Afterward, the result of the tests was reviewed and analyzed. Indeed, according to the national and in‐hospital guidelines, the type and frequency of each test were determined. The next session was dedicated to organizing and the strategic planning of the educational program and methods by the clinician in association with the residents.

Workshops for residents were held in small group and condition‐based learning. There were 15 residents in each workshop. The residents were trained about the laboratory testing guideline and test costs. The workshops were held for four consecutive days. If the residents could not attend the scheduled workshop, they could attend the next workshop.

This new guideline was published as a booklet and made available for all residents. Thus, the posters of the new guideline were provided for the obstetrics and labor ward. Also, weekly (for one month) and monthly (for 11 months) emails to all residents were made as a reminder of the protocols.

The laboratory testing orders of the residents were reviewed after 1, 3, 6, and 12 months after the workshops and the results compared before and after the educational program. The adverse events, both those resulting in perceived harm to patients and near miss of harm, were assessed. This evaluation was calculated in real‐time through polling of supervising residents on each intervention team.

### 2.2 Laboratory testing and conditions

The type and frequency of the following tests were analyzed: complete blood count (CBC), Blood Group (BG) and RH, blood urea nitrogen (BUN), Creatinine (Cr), aspartate aminotransferase (AST), alanine aminotransferase (ALT), lactate dehydrogenase (LDH), blood sugar (BS), alkaline phosphate (ALP), partial thromboplastin time (PTT), prothrombin time (PT), Fibrinogen (Fib), bilirubin (Bil), direct bilirubin (Bil‐D), and urinalysis (UA).

The diagnosis included gestational diabetes mellitus (GDM), pre‐eclampsia, preterm labor (PTL), and premature preterm rupture of membrane (PROM) as 4 main reasons for admission in the obstetrics ward as well as cesarean section (C/S) and normal vaginal delivery (NVD) in the labor ward.

### 2.3 Ethical consideration

The study was approved by local ethical committee at Tehran University of Medical Sciences in January 2019. This study was conducted according to the criteria set by the declaration of Helsinki. The Ethical code was VCR.REC.1397.252.

### 2.4 Statistical analysis

Data analysis was performed by SPSS version 19.0 software including descriptive and comparative statistics. Categorical variables were presented as numbers and percentages. We further estimated the total laboratory cost of all the tests performed in each of the two periods and costs per patient day based on the current charges of each test. The statistical significance for all outcomes was set at *p*‐value < 0.05.

## 3 RESULTS

A total of 720 patients were recruited in this study. The mean ± SD age of the patients was 29.5 ± 6.9. The main etiologies of admission in labor were cesarean section (48.8%) and normal vaginal delivery (51.2%) and in obstetrics ward were preeclampsia (35.5%); gestational diabetes mellitus (25.8%), premature preterm rupture of membrane (PPROM) (21.1%), and preterm labor (17.6%).

All 28 residents from the first year to chief residents have attended the workshop. They acted significantly better after training. The difference between their pre‐test and post‐test scores was significantly different (*p* = 0.002). The total score per patient/laboratory test drawn as 17.0 pre‐education to 7.7 post‐education.

There was a significant reduction (*p* < 0.001) except for CBC in ordering laboratory testing (Table 1). In comparison based on the diagnosis, there was no significant difference (*p* = 0.056) in ordering laboratory testing before and after intervention between various conditions.

**TABLE 1:** Change in the number of laboratory test before and after intervention

Laboratory testing	CI (minimum)	CI (maximum)	*p*
CBC	0.993	1.00	0.643
BUN	0.362	0.434	*p* < 0.001
Cr	0.362	0.434	*p* < 0.001
AST	0.362	0.434	*p* < 0.001
ALT	0.362	0.434	*p* < 0.001
LDH	0.362	0.434	*p* < 0.001
UA	0.498	0.571	*p* < 0.001
ALP	0.195	0.257	*p* < 0.001
PT	0.172	0.230	*p* < 0.001
PTT	0.172	0.230	*p* < 0.001
Bil‐D	0.172	0.230	*p* < 0.001
Bil	0.172	0.230	*p* < 0.001
Fib	0.172	0.230	*p* < 0.001
BG‐RH	0.714	0.777	*p* < 0.001
CRP	0.237	0.301	*p* < 0.001
Uric Acid	0.172	0.230	*p* < 0.001
BS	0.178	0.232	*p* < 0.001

CBC, complete blood count; BG and RH, blood group; BUN, blood urea nitrogen; Cr, Creatinine; AST, aspartate aminotransferase; ALT, alanine aminotransferase; LDH, lactate dehydrogenase; BS, blood sugar; ALP, alkaline phosphate; PTT, partial thromboplastin time; PT, prothrombin time; Fib, Fibrinogen; UA; urinalysis; Bil, bilirubin; Bil‐D, direct bilirubin; CI, confidential interval.

The cost per patient of laboratory test was decreased by 31.3% after training course and was significantly different (*p* < 0.001).

The rate of near miss or delays in the treatment of the patients was not significantly different from before the intervention (*p* = 0.457). In other words, side effects for hospitalized patients did not increase with decreasing the number of requested laboratory tests.

## 4 DISCUSSION

It is difficult to change the manner of the clinicians in requesting laboratory tests.^17^ This may due to the lack of a consensus about the appropriate laboratory testing in literature; thus, we need to set the standards for the proper laboratory testing depending on the specialty of medicine and also on the patient's clinical condition and case severity.^16^ In a study by Sedrak et al., the main cause of ordering unnecessary laboratory testing in the majority of the residents was the lack of cost transparency.^18^ This was the same as the current study, and we tried to motivate the residents to follow the guidelines by continuous monitoring.

The study by Wertheim et al.^19^ revealed that intervention produced a 9% reduction in laboratory tests request and a few delayed diagnoses or near misses were reported in the intervention arm. In our study, we detected about a 10% reduction in laboratory testing orders, but we did not detect any near miss or adverse events.

The study by Sadowski et al.^20^ showed that the first intervention resulted in up to 0.97 fewer laboratory tests per inpatient day and the second intervention led to the sustained reduction, although by less of a margin than order set alteration alone. In the current study, single level intervention had good results and it is more favorable and easier to do in a clinical setting. What was important in our study was the continuous monitoring of residents that was encouraging and further progressions were also required.

High requested laboratory such as basic metabolic panel and complete blood count may prove to be more agreeable to demand than less commonly ordered tests.^19^ Although, in our study, all orders were reduced except CBC; this may be due to the specific conditions in obstetrics.

A case study by Khalifa et al.^21^ revealed that more than 11% of requested tests in clinical inpatient settings are over‐utilized, repeated, and also unnecessary and could be deleted. The tests including CBC, renal profile, and blood sugar are responsible for 35 percent of all hospital inpatient laboratory tests. This matter was also similarly seen in our study. Thakkar et al.^22^ reported that there was significant reduction in the rate of errors in the request of the laboratory tests after intervention as same as our study.

Auditing, continuing education, and informing physicians, based on the evaluation of factors influencing irrational laboratory application, would reduce test demand.^23^, ^24^

The success of administrative work depends on the local institutional culture and strong support by hospital leadership and clinicians.^25^ As well as our study that teamwork in the hospital was formed to run this project with an association of all clinical, paraclinical, and management parts.

The involvement of residents in guideline design may improve their motivation to follow the guideline^19^ as well as our study.

One of our challenge was the resistance of the experienced professors to accept the logical request for experiments, which was solved by holding an explanatory meeting before starting the project.

Even those who have been attended the workshops forgot the list of necessary and unnecessary tests over time. Therefore, it is recommended to hold periodical workshops to reminder the necessary data. Consequently, designing a web‐based platform for laboratory test limitation and autocorrect in this stand and generalized to other hospitals is mandatory.

As we know, this study is a pioneer in obstetrics to evaluate laboratory testing. One of the benefits of this study could be the matching of laboratory testing requests in teaching and private hospitals. The strengths of this study were the cooperation and interaction between the parts of the hospital as well as educational units and the laboratory. The limitation of our study was the small sample size and a single‐center study in obstetrics. The results may not be generalizable to other institutions. Thus, we did not evaluate admitted cases and emergent conditions that may need some tests more frequently.

## 5 CONCLUSION

Totally according to the results, it may be concluded that educational programs could result in a reduction of the mistakes in some unrequired and unnecessary tests in the obstetrics and labor ward. However, further studies are required to attain more definite results for comparison with our results and a better understanding of the role of the training course for the reduction of unnecessary laboratory tests.

## CONFLICT OF INTEREST

None.

## Data Availability

The datasets generated during and/or analyzed during the current study are available from the corresponding author on reasonable request.
